# Commentary: Role of VEGF, Nitric Oxide, and Sympathetic Neurotransmitters in the Pathogenesis of Tendinopathy: A Review of the Current Evidences

**DOI:** 10.3389/fnagi.2017.00060

**Published:** 2017-03-21

**Authors:** Benjamin J. F. Dean

**Affiliations:** Nuffield Department of Orthopaedics, Rheumatology and Musculoskeletal Sciences, Botnar Research Centre, University of OxfordOxford, UK

**Keywords:** tendinopathy, inflammation, glutamates, pain, VEGF, NMDAR, tendons

I read the recent systematic review which asked some interesting questions regarding the pathogenesis of tendinopathy (Vasta et al., 2016). The statement “histologic studies have demonstrated the absence of inflammatory infiltrates” is not supported by the current evidence base. Our recent systematic review on this subject demonstrated that the absence of neutrophils does not equate to the absence of inflammatory cells (Dean et al., 2016), while several recent studies have provided compelling evidence to support the hypothesis that chronic inflammation is a key factor in the pathogenesis of tendinopathy (Dakin et al., 2015; Dean et al., 2015a). In addition the search strategy does not appear to have been comprehensive. We have carried out a number of systematic reviews in this area (Dean et al., 2016; Vasta et al., 2016) which identified numerous studies which have been missed by this review (Gotoh et al., 1998; Forsgren et al., 2005; Andersson et al., 2008; Lakemeier et al., 2010; Shindle et al., 2011; Millar et al., 2012). We have also published several pieces of work related to markers such as VEGF, glutamate, various glutamate receptors, the neurokinin-1 receptor and tyrosine hydroxylase which were also not included (Dean et al., 2014, 2015a,b; Franklin et al., 2014). It is rather problematic that so many relevant studies have not been incorporated into this systematic review. It is important that readers are fully informed of the current evidence base and thus can be made aware of the role of neuro-inflammatory change in the pathogenesis of tendinopathy.

## Author contributions

The author confirms being the sole contributor of this work and approved it for publication.

### Conflict of interest statement

The author declares that the research was conducted in the absence of any commercial or financial relationships that could be construed as a potential conflict of interest.
